# Haemato-biochemical changes and prevalence of parasitic infections of indigenous chicken sold in markets of Kiambu County, Kenya

**DOI:** 10.1080/23144599.2019.1708577

**Published:** 2020-02-13

**Authors:** Peninah Wamboi, Robert M. Waruiru, Paul G. Mbuthia, James M. Nguhiu, Lilly C. Bebora

**Affiliations:** a Department of Veterinary Pathology, Microbiology and Parasitology, Faculty of Veterinary Medicine, University of Nairobi, Kangemi-Nairobi, Kenya; b Department of Clinical Studies, Faculty of Veterinary Medicine, University of Nairobi, Kangemi-Nairobi, Kenya

**Keywords:** *Ascaridia galli*, blood glucose, ectoparasites, *Eimeria*, haematocrit, *Haemoproteus*

## Abstract

This study aimed at determining parasitic prevalence and probable haemato-biochemical changes that may occur from parasitic infections in marketed indigenous chickens in Kiambu County, Kenya. Thirty adult chickens were purchased and examined for ectoparasites, haemoparasites and haemato-biochemical changes. Post mortem was conducted to recover gastro-intestinal parasites and fecal samples taken for egg/oocyst counts. Forty-seven percent (14/30) of chickens examined were in poor body condition, 43% (13/30) in fair and 10% (3/30) in good body condition. Ectoparasites infection prevalence was 66.7% (20/30). Four haemoparasites were isolated. Overall helminths prevalence was 86.6% (26/30), nematodes at 76.7% (23/30) and cestodes at 40% (12/30). After processing fecal samples, 30% (9/30) were positive for helminth eggs and 30% (9/30) had coccidial oocysts. Relative to normal values, total erythrocyte count was low and total leucocyte count with band cells high. Mean haematocrit and heterophil values were high (p=0.0005; p=0.0061). Mean lymphocyte count was low (p=0.0128) in chickens with ectoparasitic infestation. Eosinophils increased significantly (p=0.0363) although mean erythrocytes counts decreased (p=0.0176), in chickens with gastrointestinal parasites. Creatine phosphokinase and blood glucose levels were high, serum protein and albumin levels were low. Blood glucose level decreased significantly (p=0.0239) and total plasma protein increased (p=0.045) in chickens with Haemoproteus spp. infection. The study showed, ecto- and endo-parasites are prevalent and may contribute to alteration of haemato-biochemical parameters of sub-clinically infected marketed indigenous chickens. These results are expected to contribute towards and encourage usage of clinico-pathological parameter testing as a measure of poultry health status for enhanced poultry disease diagnoses.

## 1. Introduction

Poultry, primarily chickens, are the most extensively reared livestock worldwide and the most abundant animal species [1,2]. Poultry products have been reported as some of the most vital source of protein for man worldwide [1]. The global population of poultry has been projected to be approximately 16.2 billion with 71.6% being in developing countries [3]. Within East Africa, more than 80% of human population live in rural areas, of which more than 75% keep indigenous chickens [3,4]. Kenya has an estimated population of over 37.3 million birds. Out of these, 31.578 million (84.1%) are indigenous, 3.1 million (8.4%) are layers while 2.1 million (5.7%) are broilers while, 0.522 million (1.8%) are other poultry species [5].

Indigenous chickens are mainly reared in rural areas by about 90% of the respective populace [2]. Despite the large proportion of chickens kept in rural areas, there is scarce information on rural poultry health. According to Ahmed [4], the major challenges encountered in poultry production include diseases, predation, feed shortage and scarcity of information. Concurrent disease occurrence is also a common finding in poultry. Often, bacterial, fungal and/or viral infections are accompanied by ecto- and endoparasitic infections.

Arthropod ectoparasites, like fleas and ticks have a key impact on poultry production and their welfare [6,7]. They are capable of causing severe dermatitis and allergies [8]; anaemia due to blood loss [9] and can act as vectors of pathogens causing bacterial, rickettsial and viral diseases. These diseases may cause massive loses in chickens and some are of public health importance [10,11]. Several haemoparasites infect avian species with *Plasmodium gallinaceum* being the most pathogenic leading to death in untreated cases [12]. Gastrointestinal parasitism leads to significant economic losses in poultry [13] and nematodes cause more serious problem in backyard flocks, in developing countries in Africa [14]. The backyard scavenging production system exposes chickens to arthropods and environmental conditions contaminated with eggs and larvae of parasites [15,16]. Helminth infections in rural free-range chickens are ubiquitous and may result in sub-clinical diseases even when they occur in lower numbers [17].

In Kenya, few studies have been done on ecto- and haemoparasitism of free-range poultry to date [18–20] while, literature on prevalence of gastrointestinal parasites of marketed local chickens is limited to work done by Kyalo [21] and Maina et al. [22]. Given that the prevalence of parasites vary considerably from one geographic region to another [23], it is therefore, necessary for periodic surveillance of the prevalence of these parasites within a given locality for successful formulation and implementation of an effective control strategy [24].

Haematological and biochemical parameters are good indicators of the health assessment for both animals and humans and yet they are rarely used. Sub-clinical parasitic infections contribute towards morbidity losses experienced by poultry farmers. It is, therefore, of interest to monitor changes in haemato-biochemical parameters of marketed chickens to establish their respective levels as some of these parameter-changes could be associated with parasitic infections. Limited studies have been done on assessment of clinico-pathological parameters in poultry disease diagnoses [25].

This study was designed to determine haemato-biochemical changes, which may be associated with ecto- and endo-parasites in naturally infected indigenous chickens. Results of the study are expected to contribute towards and encourage usage of clinico-pathological parameter testing as a measure of poultry health status. Accurate disease diagnosis will enable effective treatment of the disease/condition, thus contribute towards increased productivity and food security; resulting in financial empowerment and poverty alleviation for the farmers and community.

## 2. Materials and methods

### 2.1. Study area

This study was conducted in three chicken markets (Wanginge, Uthiru, Gitaru) located in Kabete and Kikuyu sub-counties of Kiambu County, Kenya. The physiographic and natural land conditions of the study area are as described by Anonymous [26].

### 2.2. Ethical approval

The Faculty of Veterinary Medicine Biosafety Animal use and Ethics Committee (BAUEC) approved this study; approval number is REF: FVMBAUEC/2018/177.

### 2.3. Sampling

Thirty adult chickens (28 females and 2 males) were randomly selected and purchased, from the three markets. An independent person numbered all the chickens in the market at the time of sampling. The investigator then mentioned a number and the chicken with that number was purchased for the study. The sample size of study chickens was determined based on the non-probability sampling technique as described by Kothari [27]. Market distribution of the birds was 12 (Uthiru), 12 (Waginge) and 6 (Gitaru). All the chickens were transported alive in cages immediately after purchase to the Department of Veterinary Pathology, Microbiology and Parasitology, University of Nairobi (UoN) where laboratory examination was conducted.

### 2.4. Determination of body condition of chickens

The method employed for body condition scoring was as described by Gregory and Robins [28]. Body condition scoring of the birds were classified from score 0–3 based on prominence of the keel bone. Good body conditioned chicken having moderately grown convex breast muscle, less prominent keel. A fair body conditioned chicken depicting more growth of breast muscle, not concave and the keel slightly protuberant. A poor body conditioned chicken had conspicuous ridge on the keel bone, scanty breast muscle with concavity along the keel [28].

### 2.5. Examination of chickens for ectoparasites

Chickens were thoroughly examined before being humanely euthanized. Ectoparasites found were processed as described by Hendrix and Robinson [29] and identified according to their morphological characteristics using entomological keys developed by Taylor et al. [12] and Wall and Shearer [30].

### 2.6. Examination of chickens for endoparasites

Birds were euthanized by cervical dislocation. Afterwards, the carotid arteries and jugular veins were severed [31]. Whole and heparinized blood was collected for serum harvesting and haematological analysis, respectively as detailed in section 2,7 below. In order to examine the haemoparasites, thin blood smears were made and examined using the blood of the jugular veins as described by Wakenell [32] and the haemoparasites, were identified on a morphological basis as described by Permin and Hansen [1] and Taylor et al. [12].

Gastrointestinal tracts of chickens were separated during post mortem into various segments (oesophagus, crop, proventriculus, gizzard, small intestines and caecum). The segments were opened separately and contents washed with water into containers making sure that the worms clinging onto the mucosa were carefully removed. The helminths collected from each chicken were separately preserved in 70% ethanol. The helminths were processed according to techniques described by Gibbons et al. [33]. For cestodes Identification was done following the procedure by Permin and Hansen [1] after preparation of stained whole mounts. Aceto alum Carmine solution was used for the general staining in order to do morphological studies. Nematodes were placed on glass slides and cleared in lactic acid for at least 30 minutes, for morphological identification. The slides were studied using light microscope with x10 and x40 objective lenses. These helminths were identified using helminthological keys of [1,12,34].

A fresh faecal sample was taken from the cloaca of each chicken and processed to determine faecal egg (nematode, cestode) and coccidial oocyst counts using a modified McMaster technique [29].

### 2.7. Analysis of haemato-biochemical parameters

Haemato-biochemical parameters were analysed at the Department of Clinical Studies, Faculty of Veterinary Medicine, UoN. The haematological parameters were analysed as described by Coles [35] and Dacie and Lewis [36], while biochemical parameters were analysed as described by Doumas [37], Bergmeyer et al. [38] and Sakas [39].

### 2.8. Data analysis

Data was entered in Microsoft office Excel version 2016 and exported to (SPSS) statistical software and for descriptive statistical analysis [40]. The prevalence of ecto- and endoparasites was defined as total number of chickens infected with the parasites divided by number of chickens examined [41]. A critical probability of 0.05 was adopted throughout as a cut-off point for statistical significance.

## 3. Results

### 3.1. Body conditions

On physical examination of the chicken, 47% were in poor body condition, 43% were fair and only 10% were in good body condition.

### 3.2. Ecto- and haemoparasites

Of the 30 chicken sampled 66.7% (20/30) had either a single or mixed infection with ectoparasites (lice or fleas). Lice had highest mono-infection prevalence out of the infected chicken at 85% (17/20) while only 15% (3/20) had co-infection of lice and fleas. Three species of lice were recovered with *Menacanthus stramineus* being the most prevalent 63.3% (19/30) followed by *Liperus caponis* 3.3% (1/30) and *Goniocotes gallinae* 3.3% (1/30). *Echidnophaga gallinacea* (stick tight flea) was the only flea recovered with a prevalence of 13.3% (4/30) (Table 1).

**Table 1.** Prevalence of ecto- and haemoparasites recovered from study chickens

Ectoparasites	No. (*n* = 30)	Prevalence (%)
**Lice**		
*Liperus caponis*	1	3.3
*Menacanthus stramineus*	19	63.3
*Goniocotes gallinae*	1	3.3
**Flea**		
*Echidnophaga gallinacean*	4	13.3
**Genera of haemoparasites**		
*Leucocytozoon*	30	100
*Haemproteus*	9	30
*Plasmodium*	30	100
*Agyeptiniella*	30	100

*Leucocytozoon* spp., *Plasmodium* spp. and *Agyeptiniella* spp. were diagnosed in all the chicken blood smears examined except *Haemoproteus* spp., which was found in 30% of the chickens (Table 1).

### 3.3. Gastrointestinal parasites

Out of the 30 chicken sampled, 86.6% (26/30) were positive for helminths and 30% were shedding *Eimeria* oocysts. Nematode species were the most prevalent helminths at 76.7% (23/30) followed by cestodes with a prevalence of 40% (12/30). Nematodes recovered were *Heterakis gallinarum* (60%; 18/30), *Ascaridia galli* (46.7%; 14/30), *Tetrameres americana* (16.7%; 5/30), *Gongylonema ingluvicola* (13.3%; 4/30), *Capillaria* spp. (6.7%; 2/30), *Allodapa suctoria* (3.3%; 1/30) and *Subulura brumpti* (3.3%; 1/30). Three cestodes were recovered and included *Raillietina echinobothrida* (33.3%; 10/30), *Choanotaenia* spp. (6.7%; 2/30) and *Hymenolepis* spp. (3.3%; 1/30). Ten of the 30 (33.3%) chicken faecal samples examined were positive for helminth parasite eggs, of which all (10/10) had *Ascaridia* spp., 40% (4/10) had *Capillaria* spp. and 10% (1/10) had cestode eggs. *Eimeria* oocysts had a prevalence of 30% (9/30).

Mixed infections occurred where the chickens were infected with more than one of the above mentioned parasites at the same time. Fifty percent of the study chickens (15/30) had triple infections with ecto-, haemo- and gastrointestinal (GI) helminths. Thirty percent (9/30) had double infection with haemo- and GI-helminths while only 10% (3/30) had a double infection with ecto- and haemoparasites. Single infections were recorded in 10% (3/30) of the study chickens that had haemoparasites only (Table 2).

**Table 2.** Prevalence of gastrointestinal (GI) parasites recovered from indigenous chickens obtained from three markets in Kiambu County

GI parasites	No. (*n* = 30)	Prevalence (%)	Predilection site
**Nematodes**			
*Heterakis gallinarum*	18	60	Caecum
*Subulura brumpti*	1	3.3	Caecum
*Gongylonema ingluvicola*	4	13.3	Esophagus/crop
*Ascaridia galli*	14	46.7	Small intestines
*Capillaria* spp.	2	6.7	Small intestines
*Allodapa suctoria*	1	3.3	Caecum
*Tetrameres americana*	5	16.7	Proventriculus
**Cestodes**			
*Raillietina echinobothrida*	10	33.3	Small intestines
*Hymenolepis* spp.	1	3.3	Small intestines
*Choanotaenia* spp.	2	6.7	Small intestines
**Protozoa**			
*Eimeria* spp.	9	30	

### 3.4. Haemato-biochemical changes

The mean haematological parameters that showed variation from normal documented range values were a decrease in total erythrocyte count at 231.20/microlitre of blood and an increase in total leucocyte count at 47.34/microlitre of blood, with presence of band cells. All the other parameters analysed were within the documented normal ranges (Table 3).

**Table 3.** Mean haematological parameter values of the study chicken

Haematological parameters	Mean values	Std. deviation	Normal reference* values
Haematocrit level (%)	35.013	3.6109	22 – 33
Total erythrocyte count (× 10^4^μl)	231.20	56.989	250 − 350
Total leucocyte count (× 10^3^μl)	47.343	30.3689	12 − 30
Lymphocytes (%)	32.17	12.946	7000 – 17,500
Monocytes (%)	15.77	8.072	150 – 2000
Heterophils (%)	40.13	15.498	3000 − 6000
Eosinophils (%)	8.40	7.596	0 – 1000
Band cell (%)	3.07	4.770	Rare
Basophil (%)	.20	.610	Rare
Thrombocyte count	36.	28	-

Key: * Reference normal values haematology values of domestic chicken [32], (%) percentage, (μl) microlitre

The biochemical parameters with elevated mean values included creatinine phosphokinase at 12113.57 IU/L and blood glucose at 309.14 mg/dl. Serum protein and albumin levels were slightly low at 5.30 g/dl and 1.88 g/dl, respectively (Table 4).

**Table 4.** Mean values of biochemical parameters that were evaluated in the blood of the 30 chicken obtained from the three study markets

Biochemical parameters	Number of samples studied	Mean values	Std. Deviation	Normal Reference* values
Blood glucose (mg/dl)	30	309.159	42.6802	197–299
Total plasma protein (g/dl)	30	6.340	1.1711	3.0–4.0
Total serum protein (g/dl)	30	5.303	1.8414	3–6 (birds)
Total serum albumin (g/dl)	29	1.883	.3129	3.28–3.8
Total serum globulin (g/dl)	29	3.162	1.0397	1.15–1.53
Serum ALT (IU/L)	29	23.13	31.449	10.6–11.9
Creatinine phosphokinase (IU/L)	30	12113.57	10338.381	100–200 (birds)

Key: * Reference normal values [63] and [35], (mg/dl) miligrams/decilitre, (g/dl) grams/decilitre, (IU/L) international units/litre

Chickens with ectoparasite infection had significantly (*p* = 0.0005) higher mean haematocrit value (36.7%) than ectoparasite-free chicken (32.4%). Heterophil value was also significantly (*p* = 0.0061) higher (43.5%) in those with ectoparasite infection relative to ectoparasite-free chicken (31%). Ectoparasite infected chickens had a significantly (*p* = 0.0128) lower mean lymphocyte level of 33.6% relative to ectoparasite-free chickens which had 39.2% (Table 5).

**Table 5.** Effect of ectoparasite presence in the study chicken on mean haematological parameters

	Ectoparasites			
Haematology parameters	Present	Absent	*t* value	*p* value
Haematocrit (%)	36.7	32.4	−3.61	0.0005*
Erythrocyte count (× 10^4^μl)	234.3	219.9	−0.89	0.3835
Leucocyte count (× 10^3^μl)	56.0	37	−1.56	0.1289
Platelet count	40	44	1.25	0.2234
Lymphocyte (%)	33. 6	39.2	2.66	0.0128*
Monocyte (%)	15.1	17.3	0.82	0.4208
Heterophil (%)	43.5	31.0	−2.97	0.0061*
Eosinophil (%)	8.1	6.7	−1.02	0.3159
Band cell (%)	3.4	4.5	1.36	0.1836
Basophil (%)	0.1	0.4	1.63	0.1137

**Key:** *Significant difference at *p* < 0.05, (%) percentage, (μl) microlitre

For GI parasite infections, significant difference (*p* = 0.0176) was found in mean erythrocyte count which was lower at 227/microlitre of blood in infected compared to GI parasite-free at 279/microlitre of blood. Eosinophil count was significantly (p = 0.0363) higher (7.63%) in those with GI parasites relative to GI parasite-free (2.67%) chickens. For *Hemoproteus* infection shown in Table 6.

**Table 6.** Effects of gastrointestinal parasites presence in the study chicken on mean haematological parameters

	Gastrointestinal parasites			
Haematology parameters	Present	Absent	*t* value	*p* value
Haematocrit (%)	35.08	34.75	−0.20	0.8457
Erythrocyte count (× 10^4^μl)	227	279.4	2.52	0.0176*
Leucocyte count (× 10^3^μl)	44.64	46.75	−0.05	0.9584
Platelet count	41	41	0.46	0.6502
Lymphocyte (%)	31.54	35	0.59	0.5582
Monocyte (%)	15.75	16	0.08	0.9385
Heterophil (%)	39.71	43.83	0.65	0.5228
Eosinophil (%)	7.63	2.67	−2.20	0.0363*
Band cell (%)	3.5	0.17	−1.72	0.0964
Basophil (%)	0.25	0.17	−0.15	0.8842

**Key:** *Significant differences at *p* < 0.05, (%) percentage, (μl) microlitre

There was a significant decrease (*p* = 0.0239) in blood glucose level (282.78 mg/dl) and a significant increase (*p* = 0.045) in total plasma protein level (6.99 g/dl) in chickens that had *Haemoproteus* spp. in their blood compared to those that were *Hemoproteus*-free (Table 7).

**Table 7.** Effects of *Haemoproteus* presence in the study chicken on mean biochemical parameter

	*Haemoproteus* spp.			
Biochemical parameters	Present	Absent	*t* value	*p* value
Blood glucose (mg/dl)	282.78	320.46	−2.39	0.0239*
Total plasma protein (g/dl)	6.99	6.06	2.10	0.045*
Total serum protein (g/dl)	5.11	5.39	−0.37	0.7157
Total serum albumin (g/dl)	1.94	1.86	0.71	0.4864
Total serum globulin (g/dl)	3.17	3.16	0.02	0.9876
Serum ALT (IU/L)	13.14	27.62	−1.15	0.2586
Creatinine phosphokinase (IU/L)	11209	12501	−0.31	0.7598

Key: * Significant difference at *p* = <0.05, (mg/dl) miligrams/decilitre, (g/dl) grams/decilitre, (IU/L) international units/litre

## 4. Discussion

In this study, ectoparasite prevalence 66.7% (20/30) was high due to lice infections 85% (17/20) than *Echidnophaga gallinacea* (sticktight flea) at 15% (3/20). Possibly because lice are host specific and thrive well in hot humid areas [42,43], unlike fleas that are not host specific and leave their host between meals [12]. However, it was unlike Nnadozie [44] in South Eastern Nigeria who reported flea dominance, while Saidu et al. [45] reported them as least occurring as this study and Adene and Dipeolu [46] did not encounter them in Western Nigeria. In Taraba State, North-Eastern Nigeria, the prevalence of lice and fleas were reported to be 15.6% and 12.5% in chickens by Gimba et al. [47]. The speculation is that these variations may be ascribed to geographic factors, climatic conditions and time of sampling during the day.

Four haemoparasite genera {*Plasmodium* spp., *Leucocytozoon* spp., *Aegyptinella* spp., 100% (30/30) and *Haemoproteus* spp. 30% (9/30)} were encountered in study chickens as three or four mixed infections as reported in previous studies in Nigeria [48] and Uganda [49]. These findings agreed with those of Poulsen et al. [50], Permin et al. [51] and Njunga [52] who recorded a frequent encounter of *Aegyptinella* spp. in birds in Africa, but are unlike Sabuni et al. [18] who did not find the parasite. *Haemoproteus* spp. was the least found parasite in this study, similar to what was reported by Sabuni et al. [18].

The present study recorded a high prevalence (86.6%) of diverse GI parasites (predominantly nematodes and cestodes) in indigenous chickens from the three markets, similar to previous reports from various regions of Kenya [19,22,53]. The high prevalence observed in these chickens may be attributed to free-range scavenging production system, where chickens are exposed to intermediate or paratenic hosts of helminths that infect poultry [15,16].

Seven nematode species have been documented and are reported in local chickens in tropical Africa [54–56] including Kenya [19,53]. *Heterakis gallinarum* and *A. galli* were predominant as reported by Ondawsy et al. [57] in Kakamega County, Kenya, Maina et al. [22] in Nairobi County found *H. isolonche, S. brumpt*, and *T. americana* were most prevalent.

In this study, three cestodes were recorded, the most prevalent one was *R. echinobothrida*; such result is comparable with those reported by Chege at al [19]. and Maina et al. [22] but differed with Ondawsy et al. [57] who encountered only *R. tetragona* which was not isolated in this study. In East Africa, Permin et al. [54] have reported presence of 10 species of tapeworms. Trematodes were not observed in this study, just like in Kenya [19], Tanzania [58] and Nigeria [47], however *Echinostoma revolutum* has been recorded in Kenya [21,57], Algeria [59] and Uganda [60]. *Eimeria* spp. prevalence was 30%, as previously reported at 25.6% in various agro-climatic zones of Kenya by Kaingu et al. [61]. Gimba et al. [47] recorded a lower prevalence (13.2%) in village chickens in North-Eastern Nigeria.

Ecto- and endo-parasites in the study chickens could have contributed to poor body condition, by lowering feed efficiency, feed competition and possibly affected their health. This could lower income generation and nutritional status of the rural households [17,47]. Thus, farmers stocking free-range chicken from markets as a source are advised to undertake clinico-pathological diagnosis, quarantine, treat and vaccinate appropriately prior to mixing with existing flocks [62].

There was a decrease in total erythrocyte count and an increase in total leucocyte count compared with normal values, as reported by Wakenell [32]. The variations may be attributed to parasitism, diseases, chicken breed, nutrition status, hormones and climatic conditions as reported by Campbell [63]. Total erythrocyte count decreased significantly (*p* = 0.0176) with GI parasite infections as reported by Deka and Borah [64] with *A. galli* infection which causes mild/acute enteritis, hinders vital nutrients absorption and lowers erythropoiesis. There was significant increase in eosinophils (*p* = 0.0363) an indication of parasitic infection [65], and as reported by Deka and Borah [64].

Haematocrit was significantly high (*p* = 0.0005) in chickens infected with ectoparasites; this may be due to heamoconcetration polycythaemia due to body fluid loss from parasitism although there is no clear explanation as reported by Al-Saffar and Al-Mawla [66]. The high white blood cell counts (predominantly by heterophils) may be an indicator of parasitism, nutritional and environmental factors that can trigger leukocytosis [67]. Heterophils are involved in phagocytosis of any foreign body or dead tissue, increased significantly (*p* = 0.0061) in all infected chickens as previously observed by Al-Saffar and Al-Mawla [66] but lymphocytes decreased significantly (*p* = 0.0128) in the affected chickens similar to Charles-Smith et al. [68].

Mean creatinine phosphokinase and blood glucose were elevated but serum protein and albumin levels were low than normal values [14]. For chickens with *Haemoproteus* infection, blood glucose was lowered significantly (*p* = 0.0239). Decreased glucose level in this case may be due to utilization of blood glucose by the haemoparasites present for their metabolic maintenance within the blood. Total protein increased significantly (*p* = 0.045) in chickens infected with *Haemoproteus* spp. as reported in crows with haemoparasites infection which had elevated plasma protein and globulin levels [64]. There is a need for controlled studies to elucidate the association of blood biochemical parameters and haemoparasites infection in domestic chickens.

## 5. Conclusions

Present findings suggest that apparently healthy indigenous chickens sold in selected markets in Kiambu County, Kenya are carriers of various ecto- and endoparasites and infections are mostly sub-clinical. These parasitic infections may alter haemato-biochemical parameters, which would be of diagnostic value in naturally infected poultry. Integrated control strategies are indicated to improve the health and productivity of indigenous chicken.

It would therefore, be recommended that chicken purchased at markets for rearing or restocking be isolated and treated appropriately to avoid introduction of infection and infestation to already existing flocks in the farm. Further research should be carried out to determine the effects of parasites on haemato-biochemical parameters.
